# Molecular design of the phenol type extractants

**DOI:** 10.1186/2193-1801-2-120

**Published:** 2013-03-20

**Authors:** Sergey A Semenov, Aleksandr M Reznik

**Affiliations:** Lomonosov Moscow State University of Fine Chemical Technology, prospect Vernadskogo, 86, Moscow, 119571 Russia

**Keywords:** Molecular design, Extrаctants of phenolic type, Desirable function, Quantum chemistry

## Abstract

A method of optimisation of new extractants structure using the desirable function has been developed. Earlier the desirable function has been proposed by Harrington (Ind Qual Control 21: 494-498, 1965) for the optimisation of processes with several response functions. The developed method of optimisation of new extractants structure has been used for construction of phenolic type extractants (PTE) (a class of N-(2-hydroxy-5-nonylbenzil)-dialkylamines). It has been offered to use the charge on the nitrogen atom, the heat of dissociation of phenolic group, the logarithm of distribution factor of extractant between water and octanol (computed data) and maximum permissible concentration of extractants in aqueous phase (MPC) of the o-replaced phenols (the literary data) as the controllable parameters, defining efficiency of extractants for molecular design of PTE. During optimisation of extractants structure the quantity of alkyl substitutes at nitrogen atom, the carbon atoms number in these substitutes and the electronegative substitutes in o-position to phenolic group have been varied. As the result of the molecular design, the optimal structure of PTE found is N-(2,3-dihydroxy-5-nonylbenzil)-didecylamine, which perfectly meets the requirements to industrial extractants.

**Electronic supplementary material:**

The online version of this article (doi:10.1186/2193-1801-2-120) contains supplementary material, which is available to authorized users.

## Introduction

The search and development of new effective extractants are an important scientific and practical issue (Zolotov 1982). There are some requirements to the extractant for metal extraction when using the solvent extraction method. However in practice, it is difficult to find an extractant responsible for all requirements, so one usually compromises and chooses a solution (Ritcey & Ashbrook 1979). The testing of substances, used in other industries as extractants, is of big importance in the expansion of the range of extractants for the extraction of metals (Zolotov 1982). We suggest the use alkyl derivatives of phenols, applied in synthetic rubber industry, plastic industry and elastomers industry, as extractants (Bukin et al. 1999; Bychenkov et al. 2008; Gladikova et al.^2002^). However, despite the promise of this idea, it is necessary to consider the fact, that reagents developed for other purposes may not meet to all requirements for extractants. That is why the problem of extractant molecule design, that meets the certain requirements, arises. The computer technology development in recent times allows solving this problem by using calculation methods, like the methods of quantum chemistry or the method of group contributions, etc. The development of a new, more effective extractant allows to reduce expenditures for the rare metals extraction and opens new perspectives in the certain metals usage, that are in limited use because of their high price (for example, scandium) (Korshunov et al. 1987; Komissarova 2006).

In the works (Hay 2008; Varnek 2008) in the course of the molecular design of extractants thermodynamic criteria are being considered: the energy of interaction between the receptor−ion (Hay 2008) and the constants of solvent extraction, distribution coefficients and separation coefficients of extractable metals (Varnek 2008), with the non thermodynamic factors such as MPC (maximum permissible concentration) are not taken into account.

The purpose of the current investigation is the development of a method for optimisation the structure of new extractants using the desirability function, proposed earlier by Harrington (1965) for the optimisation of processes, characterised by a few response functions (Ahnazarova & Kafarov 1978).

According to Harrington, the desirability function is a dimensionless scale, which allows to convert any response so that it is interpreted in the terms of usefulness or desirability for any specific application.

For the unilateral restrictions like *y* ≤ *y*_*max*_ or *y* ≥ *y*_*min*_ (*y*−response function) a suitable form of the transformation *y* in *d* (particular desirability function) is the exponential function:

1 $$d=\text{exp}\left[-\text{exp}\left(-y'\right)\right]$$

where:

2 $$y'=b_{0}+b_{1}y$$

The coefficients *b*_*0*_ and *b*_*1*_ will be determined, if for the two values of given property *y* one sets the corresponding values of the desirability *d*, preferably in the range 0.2 < *d* < 0.8.

Having a few responses, converted into *d* scale, it is possible to combine the generalised desirability index *D* from the different *d*, using the following expression:

3 $$D=\;k\sqrt{d_{1}d_{2}\ldots \;d_{k}}$$

## Methodology

The proposed method of the optimisation the structure of new extractants has been used for the designing phenolic type extractants (PTE) (class N-(2-hydroxy-5-nonylbenzil)-dialkylamines). Moreover the following controllable parameters were selected:

1. The formal charge on the nitrogen atom (*q*), as some metals such as scandium (Bychenkov et al. 2008; Gladikova et al. 2002) are extracted by PTE with the formation of chelates and the nitrogen atom inclusion to the chelate cycle. The value of *q* was calculated by the Milliken approach with the DFT (B3LYP) method, 6-21G basis set using the GAMESS-US (version September 7, 2006) program package.
2. The logarithm of the partition coefficient for n-octanol/water (log*P*), calculated by means of the group contribution method using the program Chem3D Ultra version 7.0.0, included in the package of applied programs (PAP) ChemOffice Ultra 7.0.1. The value log*P* allows estimating the solubility of the extractant into the aqua phase and, therefore, the possible losses of the extractant during the solvent extraction process.
3. The energy of dissociation of the phenolic group (Δ*E*_*d*_), calculated by the DFT (B3LYP) method, 6-21G basis set using the GAMESS-US (version September 7, 2006) program package. According to (Gladikova et al. 2002), in the course of scandium solvent extraction by the N-(2-hydroxy-5-nonylbenzil)-β,β-dihydroxyethylamine (NBEA) extractant the optimum pH is 4.5. However, in industrial products containing scandium there is a large amount of iron (III) as a rule (Korshunov et al. 1987), and during the precipitation of iron hydroxide large losses of scandium take place due to co precipitation. So, the problem of PTE designing, which extracts scandium at lower pH, arises. Therefore, the choice of given option is specified by the necessity of increasing the acidity of the phenolic group by introducing electronegative substituents in the ortho-position to it (Nesmeyanov & Nesmeyanov 1970).
4. MPC of o-substituted phenols (Chernyshev et al. 1999; Chernyshev et al. 2004; Chernyshev et al. 2005).

**Figure 1:**
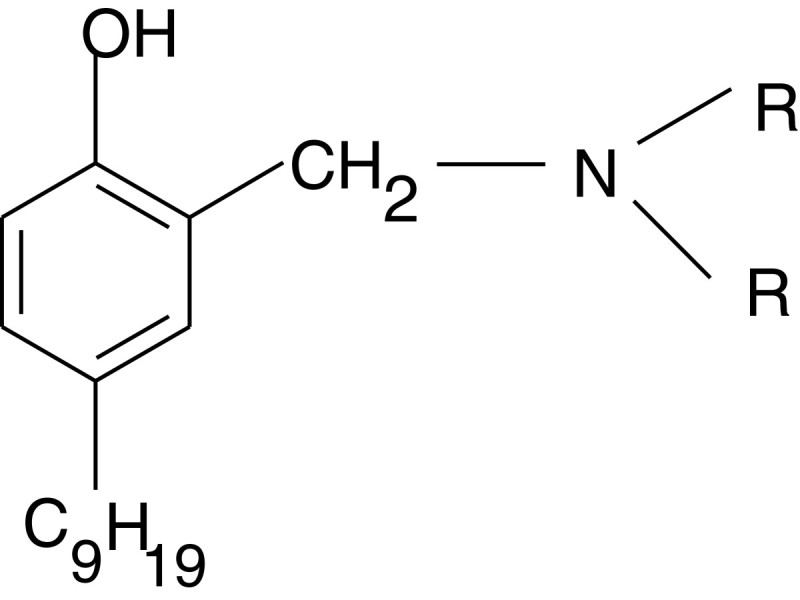
**N-(2-hydroxy-5-nonylbenzil)-dialkylamine (NBAA); R = C**_**n**_ **H**_**2n+1**_ **.**

As an initial structure for further optimisation N-(2-hydroxy-5-nonylbenzil)-dialkylamine (NBAA) was selected (Figure 1).

Earlier, the representatives of this class of extractants – NBEA and N-(2-hydroxy-5-nonylbenzil)-β-hydroxyethylamine (NBEA-1), which are used in the synthetic rubber industry, were tested for scandium solvent extraction.

## Results and discussion

At the first stage of investigation with the purpose of extractant structure optimisation the length of hydrocarbon substituents *R* was varied at the nitrogen atom. The maximum length of the hydrocarbon radical was limited to ten carbon atoms in order to avoid the high viscosity of the extractant, the decrease of its capacity of extracted component and the increase of the steric hindrances of complexation (Rozen & Krupnov 1996). The results are shown in Table 1.

**Table 1:** **The values of** ***q*** **and log** ***P*** **for NBAA extractants at varying lengths of two hydrocarbon radicals**

Extractant	***q***	log***P***
NBAA-00, n = 0	−0.093	5.11
NBАА-11, n = 1	−0.403	5.99
NBАА-22, n = 2	−0.449	7.05
NBАА-33, n = 3	−0.443	8.11
NBАА-44, n = 4	−0.440	9.16
NBАА-55, n = 5	−0.436	10.22
NBАА-77, n = 7	−0.428	12.34
NBАА-1010, n = 10	−0.456	15.51

As can be seen in the Table 1, the hydrocarbon radical length increasing both the charge of the nitrogen atom (in absolute value) and the logarithm of the distribution of NBAA between the aqua and octanol rises. The increasing of the charge at the nitrogen atom leads to rise in extraction ability of this extractant, and an increase of log*P* shows a decrease of its solubility in aqueous phase. Obtained results are consistent with the data given in (Rozen & Krupnov 1996). Consequently, in the first stage of optimisation NBAA-1010 was the most effective extractant.

**Figure 2:**
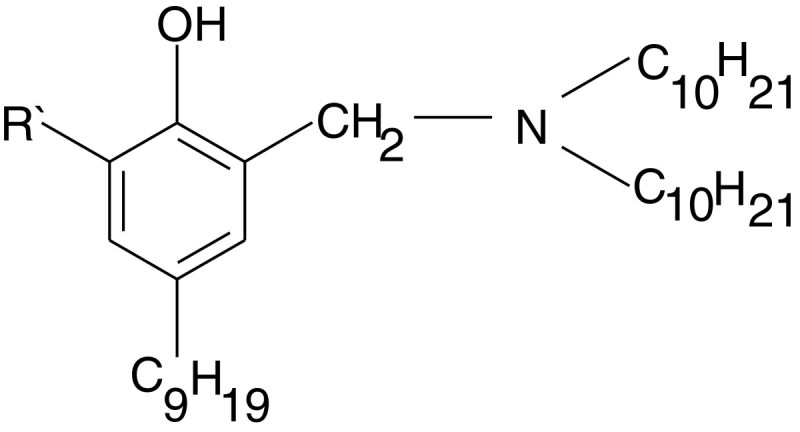
**The substituted in the** ***o*** **-position N-(2-hydroxy-5-nonylbenzil)-didecylamine.**

In the second stage of the structure optimisation of phenol type extractants by varying of the substituents in the ortho-position to the phenolic group *o*-substituted N-(2-hydroxy-5-nonylbenzil)-didecylamine was used as the original compound (Figure 2).

The acid dissociation constant of the phenolic group was evaluated by the *o*-substituted phenols dissociation energy, which was calculated by DFT (B3LYP) method, 6-21G basis set.

The calculated values of the dissociation energy Δ*E*_*D*_ for NBDA-R’, the charge on the nitrogen atom and the logarithm of the distribution coefficient between the aqua and octanol are shown in Table 2. It is obvious that the charge on the nitrogen atom changes to a small extent probably due to the remoteness of the electronegative substituent relatively the nitrogen atom, and the logarithm of the distribution index by the transition from the nitro-group to the iodide ion increases, which is consistent with the decrease of the hydrophilicity of these substituents.

**Table 2:** **The dissociation energy Δ** ***E***_***D***_ **, the charge on the nitrogen atom and log** ***P*** **of NBDA- R’ extractants**

Extractant	Δ ***E***, a.u.	***q***	log***P***
NBDА − OH	0.598	−0.285	14.9
NBDА − NO_2_	0.551	−0.279	13.8
NBDА − F	0.582	−0.278	15.8
NBDА − Cl	0.587	−0.294	16.2
NBDА − Br	0.583	−0.294	16.4
NBDА − I	0.576	−0.279	16.6

An important indicator, determining the possibility of the extractant use in rare metal technology, is the maximum permissible concentration (MPC) of extractant in the aqueous phase. Considering the solubility of extractant in the aqueous phase and its losses in the formation of an emulsion, one should always take into account MPC of the extractant when selecting. During the study of literature only 4 MPC values were found, namely: for phenol, *o*-chlorophenol, *o*-nitrophenol and *o*-hydroxyphenol (pyrocatechol) (Table 3).

**Table 3:** **The values of o-substituted phenols MPC**

Substitutes in ortho-position to phenolic group	MPC_w_(in aqua phase), mg/m^3^
without R’ (Chernyshev et al. 2005)	0.001
-Cl (Chernyshev et al. 1999)	0.0001
-NO_2_ (Chernyshev et al. 2004)	0.06
-OH (Chernyshev et al. 2004)	0.1

In solving the PTE optimisation problem as responses one used: *y*_*1*_ = *q* – the charge on the nitrogen atom, *y*_*2*_ = log*P* – logarithm of the PTE distribution index between water and octanol; *y*_*3*_ = Δ*E*_D_ – the energy of the phenol group PTE dissociation, a.u.; *y*_*4*_ = MPC_w_ - the maximum permissible concentration in water, mg/m^3^; *D-* the generalised desirability function.

For the comparative evaluation of the extractants effectiveness with various substituents in *o*-position to the phenolic group the generalised desirability function was determined by the formula:

4 $$D_{4}\;=\;4\sqrt{d_{1}\;\times \;d_{2}\;\times \;d_{3}\;\times \;d_{4}},$$

where *d*_*1*_*, d*_*2*_*, d*_*3*_*, d*_*4*_ – particular desirability functions.

It is necessary to convert obtained PTE characteristics into dimensionless uniform scale *у*^*′*^, to construct particular desirability functions. The developed extractant is to meet the specified requirements by the four indicators. Based on these requirements the values *y*_*1*_*, y*_*2*_*, y*_*3*_*, y*_*4*_, corresponding to two base benchmarks on the desirability scale, were selected.

The response *y* into the desirability function *d* was converted by the Equation (**1**). The results are given in Table 4.

**Table 4:** **The calculation of the generalised desirability function**

Substitutions in o-position	Particular desirability functions				Generalised desirability functions	
	***d***_***1***_( ***q***)	***d***_***2***_(log ***P***)	***d***_***3***_***(ΔE***_***D***_)	***d***_***4***_(MPC_W_)	***D***_3_^*)^	***D***_4_^**)^
Without the substituent	0.690	0.689	0.400	0.290	0.579	0.476
-NO_2_	0.430	0.700	0.500	0.500	0.494	0.505
-F	0.400	0.726	0.560	-	0.546	-
-Cl	0.700	0.771	0.530	0.250	0.659	0.495
-Br	0.690	0.792	0.550	-	0.669	-
-I	0.410	0.810	0.590	-	0.581	-
-OH	0.530	0.600	0.460	0.650	0.527	0.541

* *D*_*3*_ was calculated by the equation $D_{3}\;=\;3\sqrt{d_{1}\;\times \;d_{2}\;\times \;d_{3}}.$

** *D*_*4*_ was calculated by the equation $D_{4}\;=\;4\sqrt{d_{1}\;\times \;d_{2}\;\times \;d_{3}\times \;d_{4}},\;$

**Figure 3:**
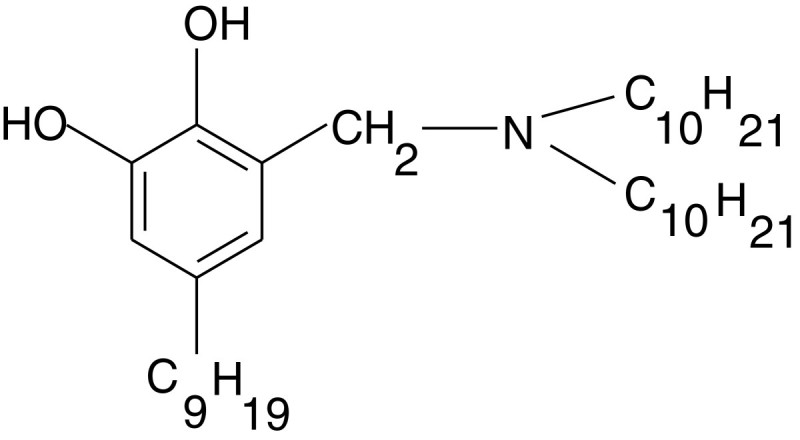
**N-(2,3-dihydroxy-5-nonylbenzil)-didecylamine.**

Table 4 shows that the extractant with bromine substituent in the o-position to the phenolic group has the maximum value of the desirability function by three criteria (the charge on the nitrogen atom, logarithm of the PTE distribution index between water and octanol; the energy of the phenolic group dissociation). N-(2,3-dihydroxy-5-nonylbenzil)-didecylamine is the most effective extractant by the four criteria (besides the three above mentioned, also the MPC value in the aqueous phase) (Figure 3).

## Conclusions

Eventually, as a result of the molecular design of the extractant there have been found the optimal structure, that is N-(2,3-hydroxy-5-nonylbenzil)-didecylamine, which meets to the maximum extent to the considered requirements to the industrial extractants. We plan to introduce the proposed extractant into the technology of scandium, rhenium, gallium, cobalt and other rare metals extraction after the synthesis and experimental verification.

## Nomenclature

d  Particular desirability function

D  generalised desirability index

logP  the logarithm of the partition coefficient for n-octanol/water

q  formal charge on the nitrogen atom

y  response function

## Authors’ original submitted files for images

Below are the links to the authors’ original submitted files for images. Authors’ original file for figure 1 Authors’ original file for figure 2 Authors’ original file for figure 3
