# Supplementary figures and images for: Molecular design of the phenol type extractants
Source: Springerplus. 2013 Mar 20;2(1):120. doi: 10.1186/2193-1801-2-120 (PMC3610026; doi:10.1186/2193-1801-2-120)

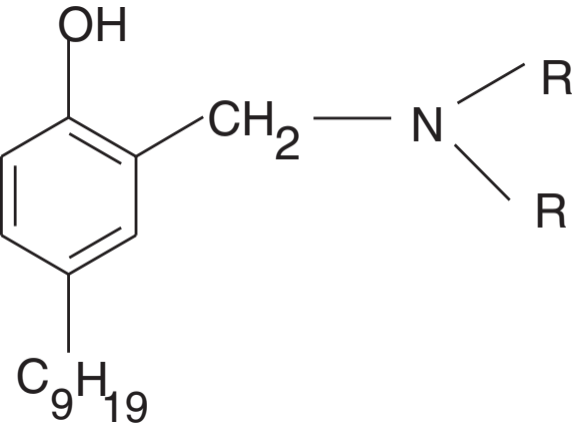

Supplement: Supplementary file 1 — Authors’ original file for figure 1 [file 40064_2013_174_MOESM1_ESM.pdf]

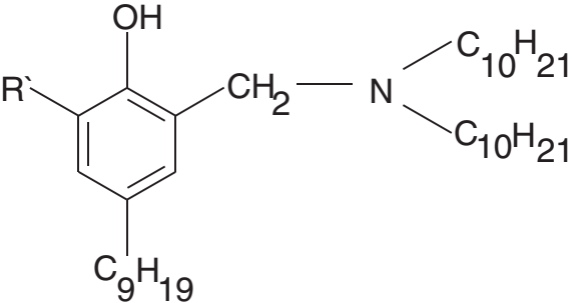

Supplement: Supplementary file 2 — Authors’ original file for figure 2 [file 40064_2013_174_MOESM2_ESM.pdf]

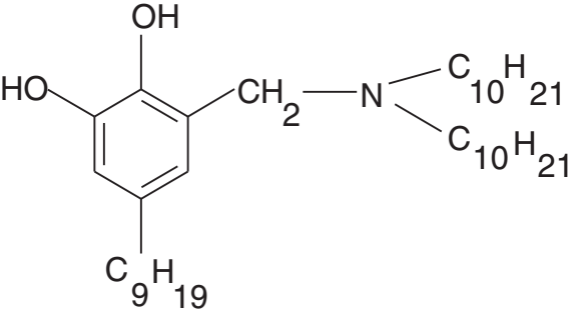

Supplement: Supplementary file 3 — Authors’ original file for figure 3 [file 40064_2013_174_MOESM3_ESM.pdf]
